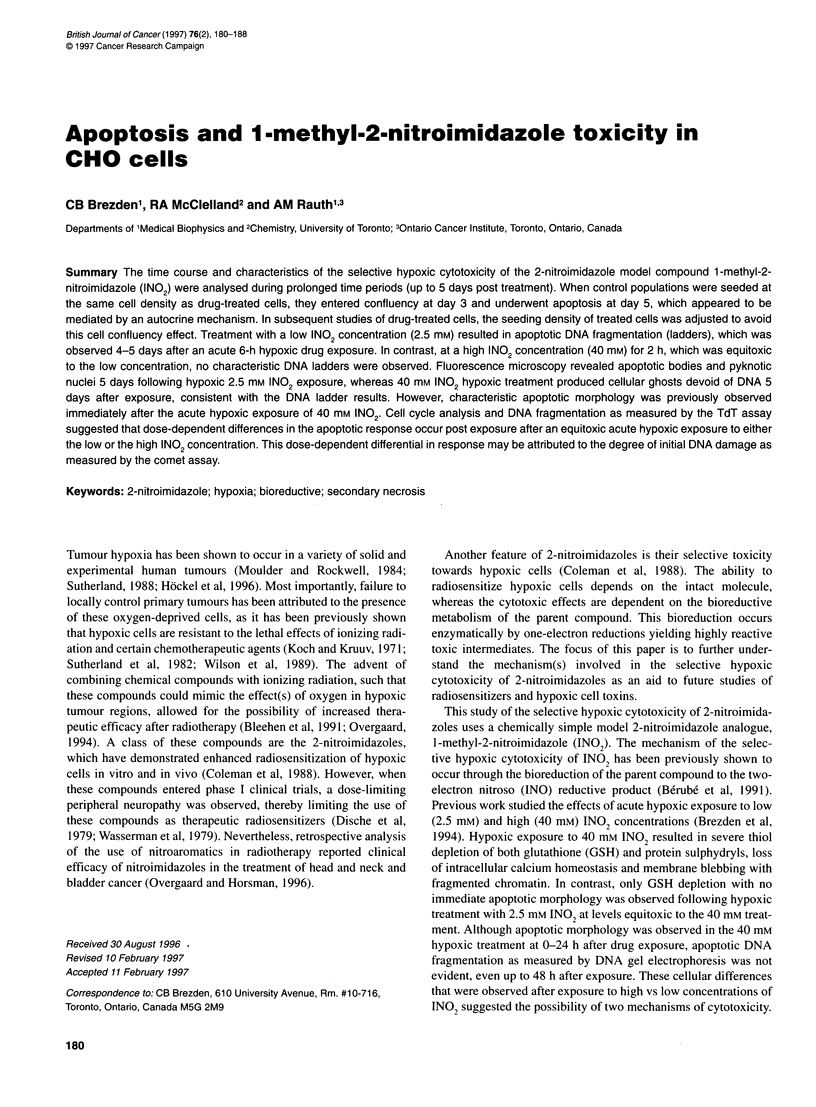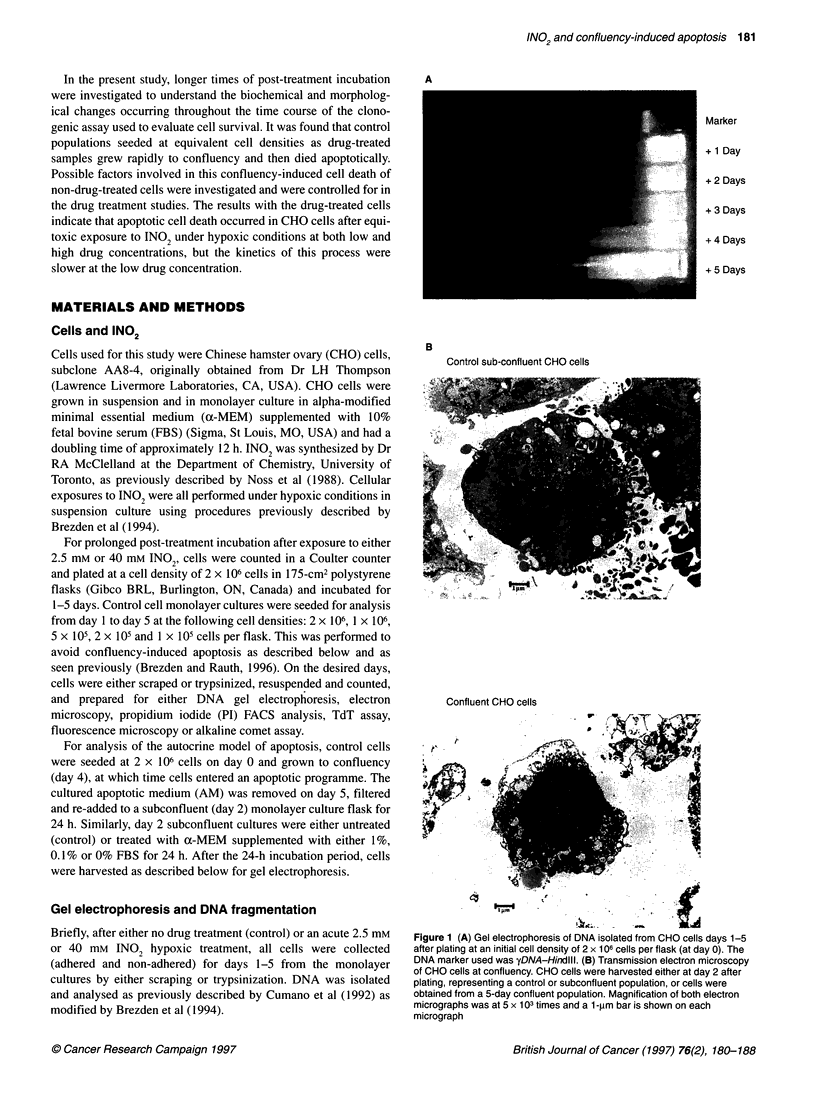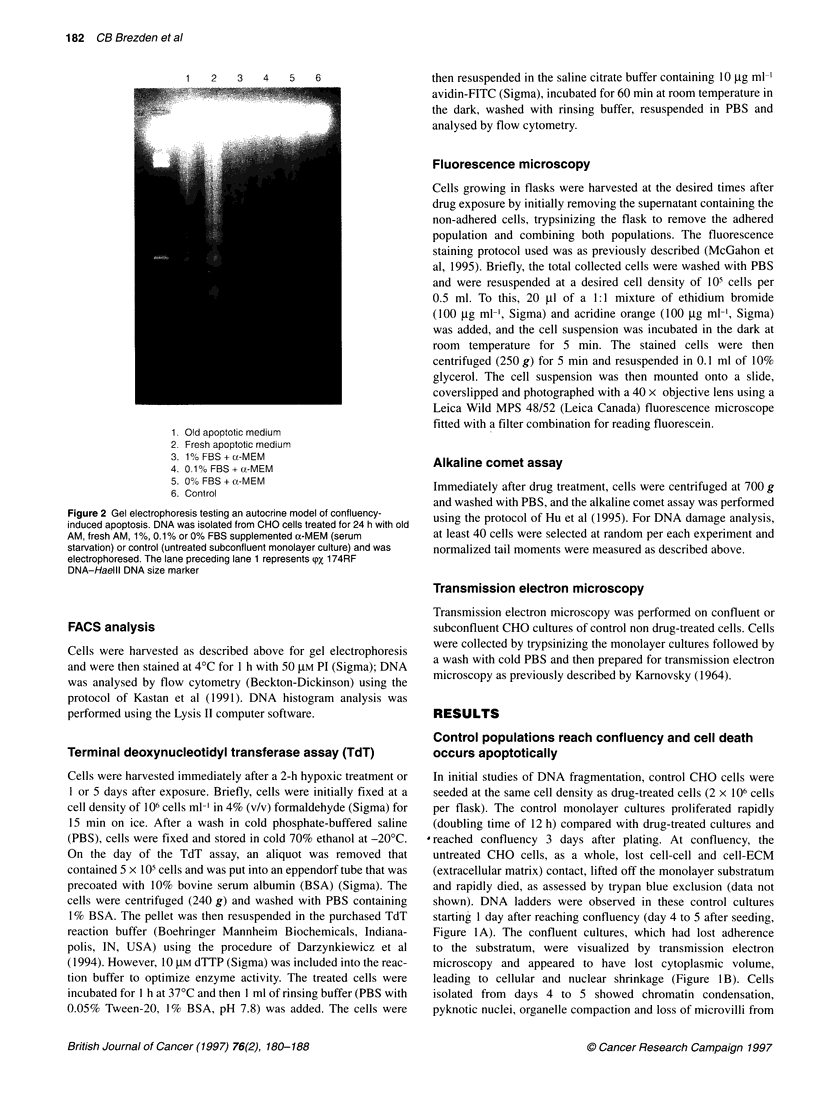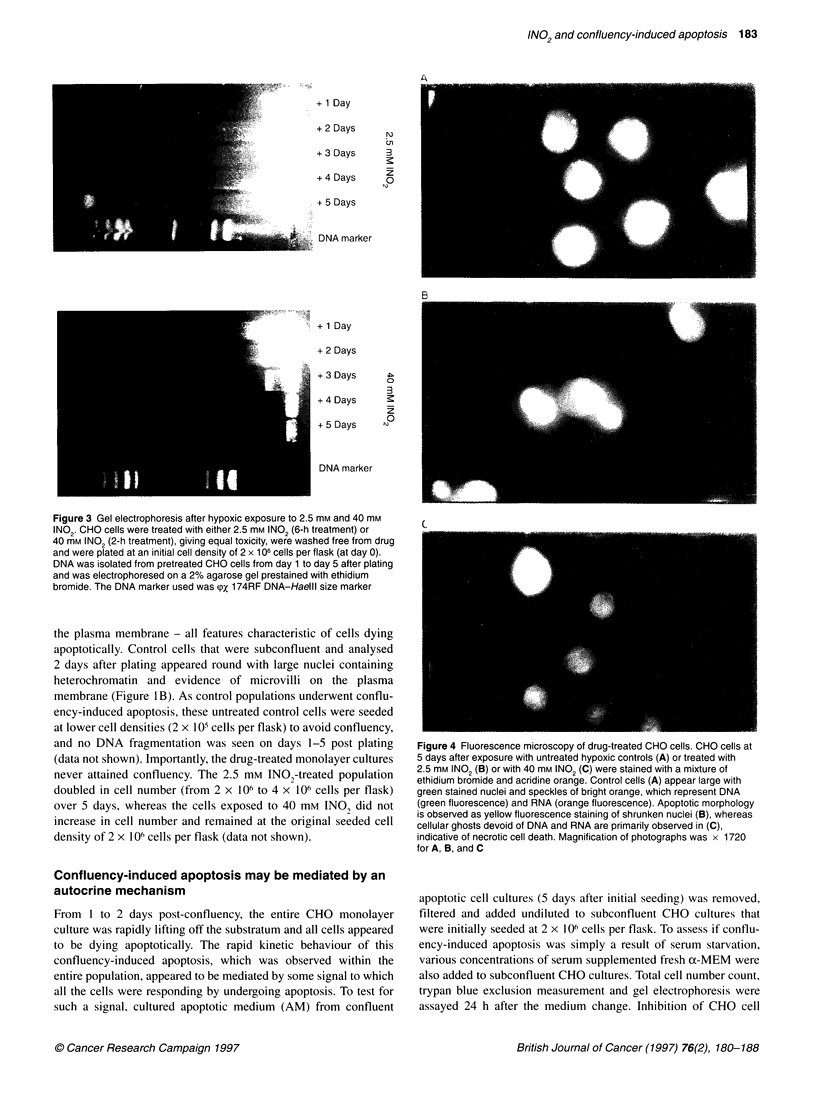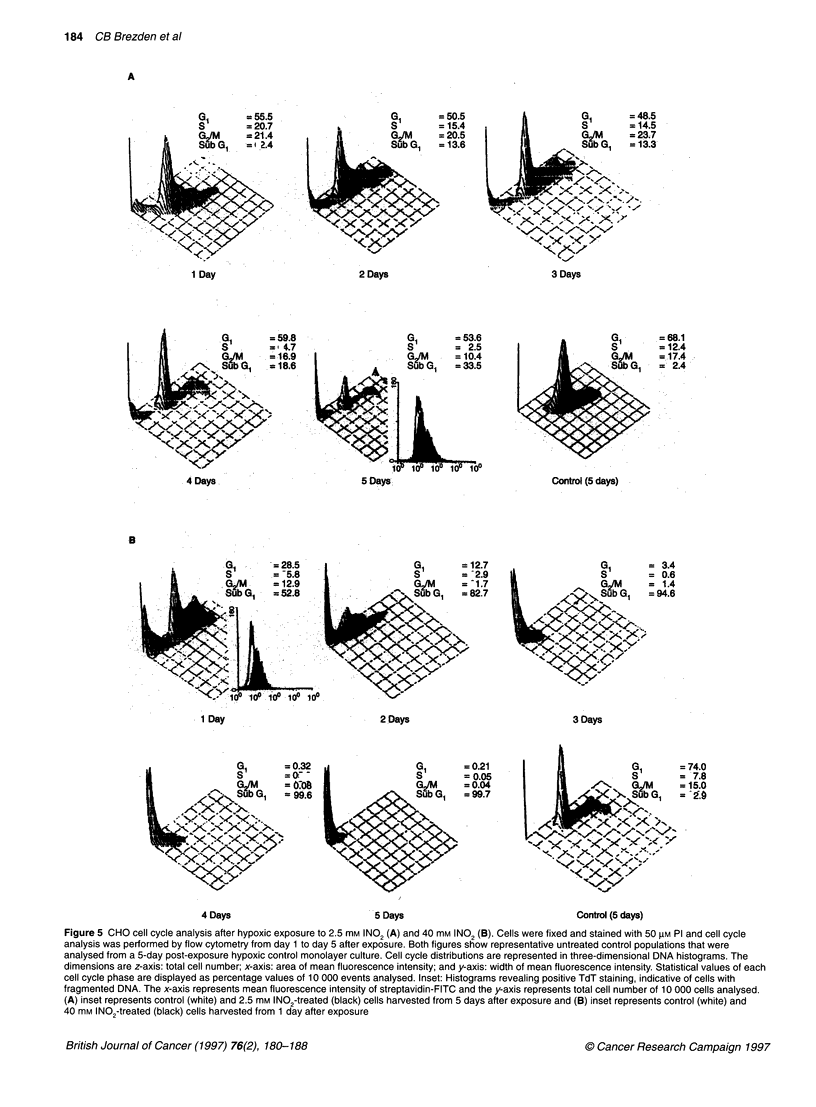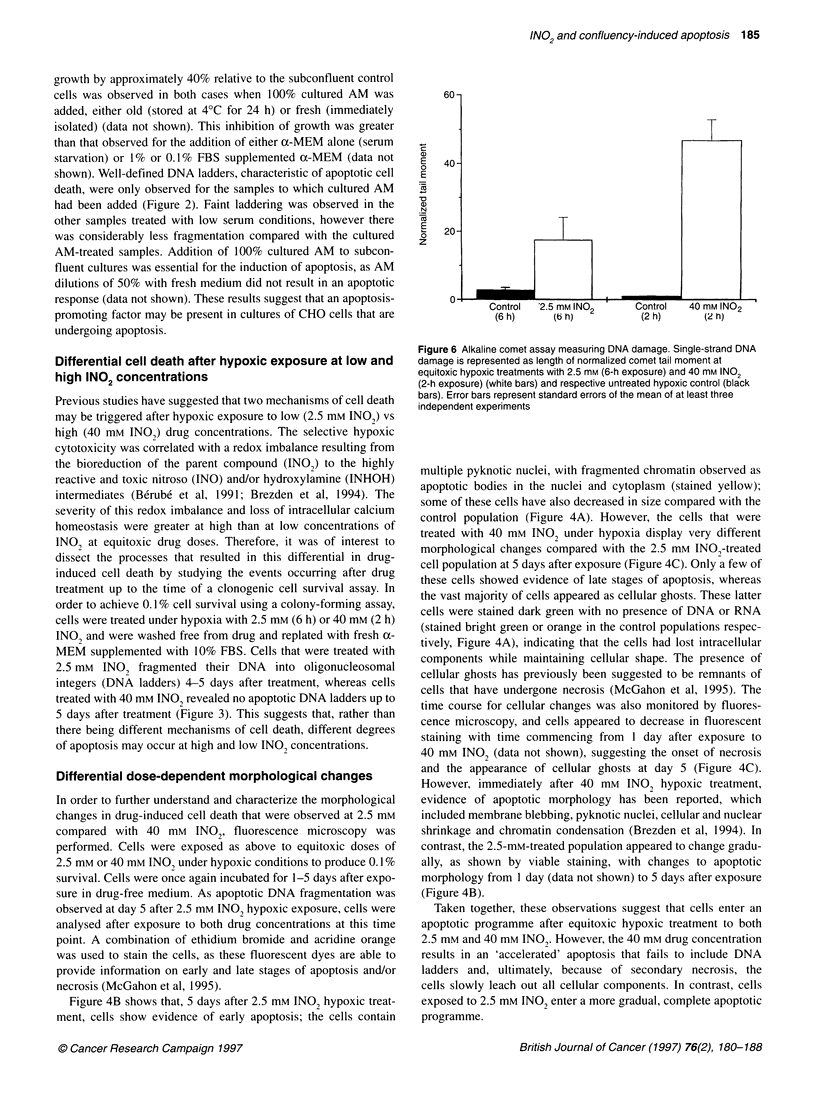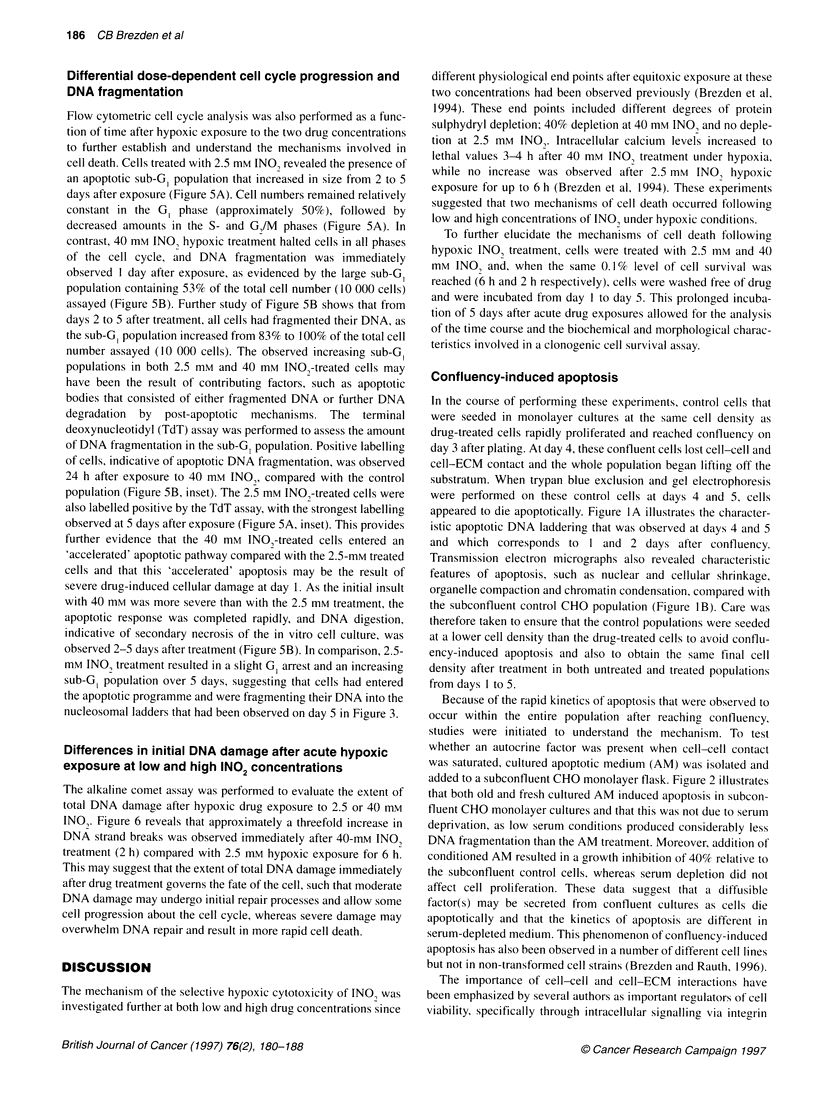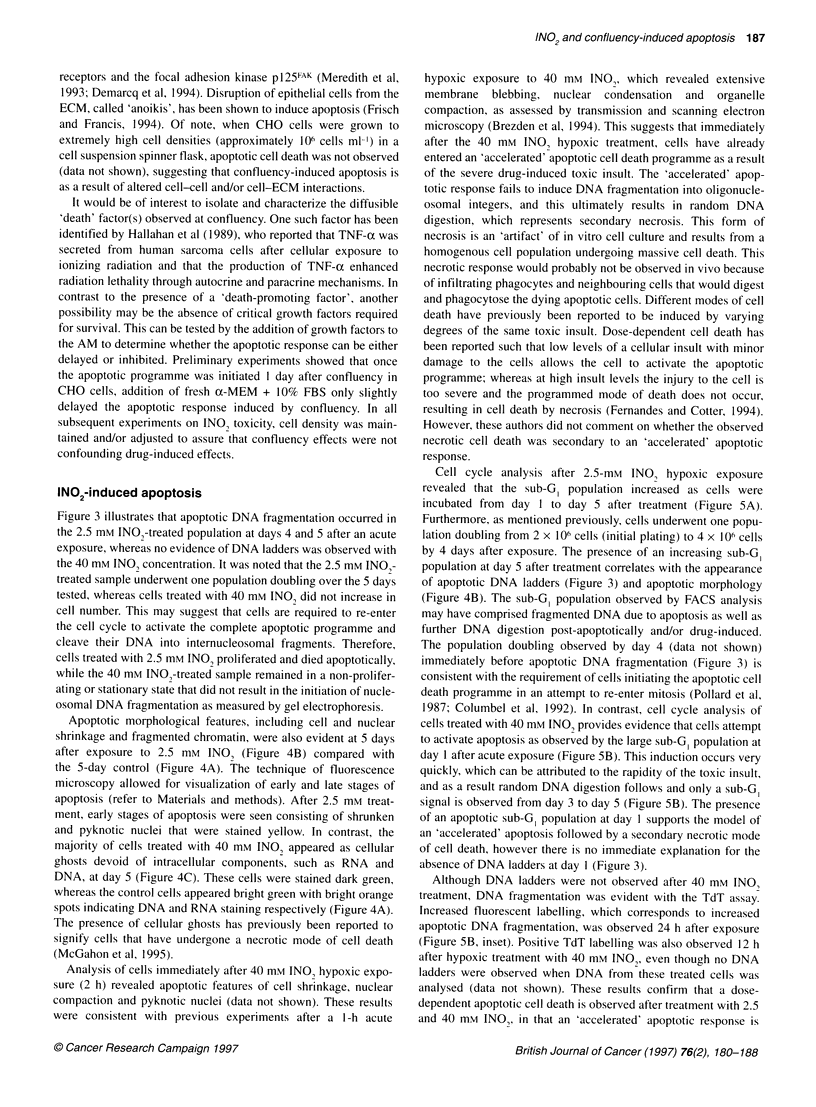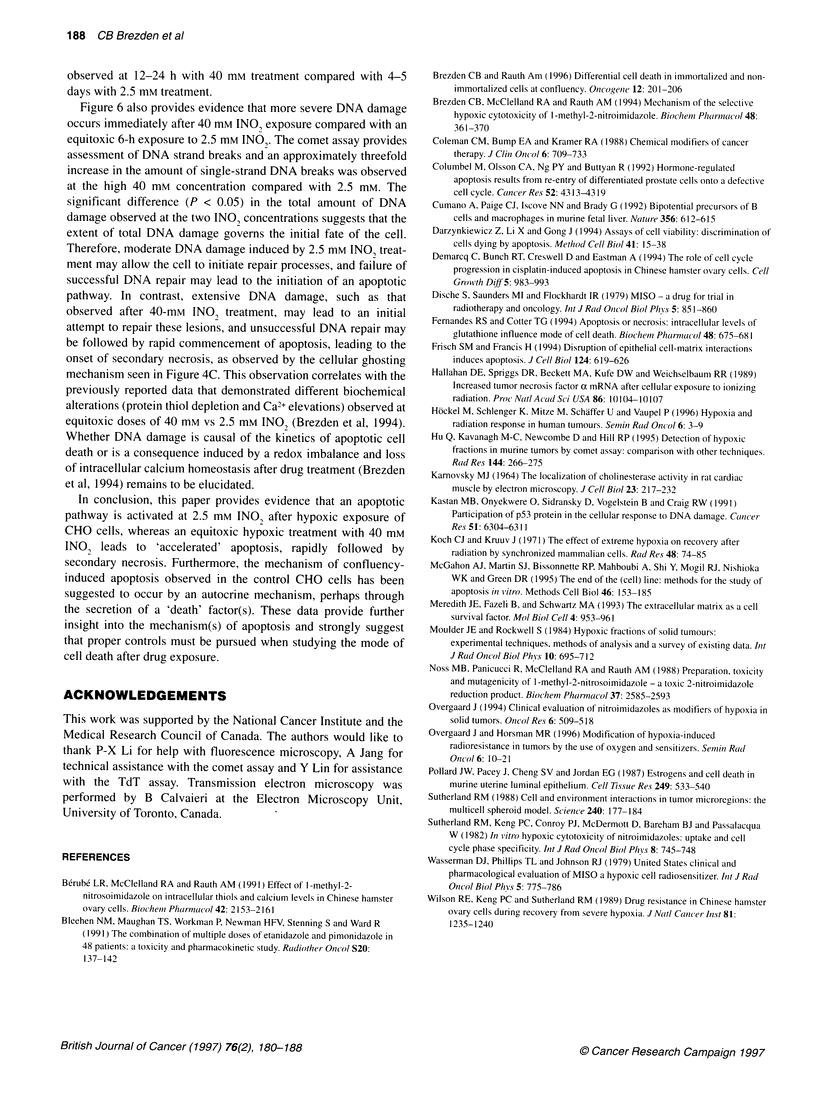# Apoptosis and 1-methyl-2-nitroimidazole toxicity in CHO cells.

**DOI:** 10.1038/bjc.1997.360

**Published:** 1997

**Authors:** C. B. Brezden, R. A. McClelland, A. M. Rauth

**Affiliations:** Department of Medical Biophysics, University of Toronto, Ontario, Canada.

## Abstract

**Images:**


British Journal of Cancer (1997) 76(2), 180–188
© 1997 Cancer Research Campaign

### Apoptosis and 1-methyl-2-nitroimidazole toxicity in CHO cells

**CB Brezden[1], RA McClelland[2] and AM Rauth[1,3]**

Departments of [1]Medical Biophysics and [2]Chemistry, University of Toronto; [3]Ontario Cancer Institute, Toronto, Ontario, Canada

**Summary** The time course and characteristics of the selective hypoxic cytotoxicity of the 2-nitroimidazole model compound 1-methyl-2-nitroimidazole ($INO_2$) were analysed during prolonged time periods (up to 5 days post treatment). When control populations were seeded at the same cell density as drug-treated cells, they entered confluency at day 3 and underwent apoptosis at day 5, which appeared to be mediated by an autocrine mechanism. In subsequent studies of drug-treated cells, the seeding density of treated cells was adjusted to avoid this cell confluency effect. Treatment with a low $INO_2$ concentration (2.5 mM) resulted in apoptotic DNA fragmentation (ladders), which was observed 4–5 days after an acute 6-h hypoxic drug exposure. In contrast, at a high $INO_2$ concentration (40 mM) for 2 h, which was equitoxic to the low concentration, no characteristic DNA ladders were observed. Fluorescence microscopy revealed apoptotic bodies and pyknotic nuclei 5 days following hypoxic 2.5 mM $INO_2$ exposure, whereas 40 mM $INO_2$ hypoxic treatment produced cellular ghosts devoid of DNA 5 days after exposure, consistent with the DNA ladder results. However, characteristic apoptotic morphology was previously observed immediately after the acute hypoxic exposure of 40 mM $INO_2$. Cell cycle analysis and DNA fragmentation as measured by the TdT assay suggested that dose-dependent differences in the apoptotic response occur post exposure after an equitoxic acute hypoxic exposure to either the low or the high $INO_2$ concentration. This dose-dependent differential in response may be attributed to the degree of initial DNA damage as measured by the comet assay.

**Keywords:** 2-nitroimidazole; hypoxia; bioreductive; secondary necrosis

Tumour hypoxia has been shown to occur in a variety of solid and experimental human tumours (Moulder and Rockwell, 1984; Sutherland, 1988; Höckel et al, 1996). Most importantly, failure to locally control primary tumours has been attributed to the presence of these oxygen-deprived cells, as it has been previously shown that hypoxic cells are resistant to the lethal effects of ionizing radiation and certain chemotherapeutic agents (Koch and Kruuv, 1971; Sutherland et al, 1982; Wilson et al, 1989). The advent of combining chemical compounds with ionizing radiation, such that these compounds could mimic the effect(s) of oxygen in hypoxic tumour regions, allowed for the possibility of increased therapeutic efficacy after radiotherapy (Bleehen et al, 1991; Overgaard, 1994). A class of these compounds are the 2-nitroimidazoles, which have demonstrated enhanced radiosensitization of hypoxic cells in vitro and in vivo (Coleman et al, 1988). However, when these compounds entered phase I clinical trials, a dose-limiting peripheral neuropathy was observed, thereby limiting the use of these compounds as therapeutic radiosensitizers (Dische et al, 1979; Wasserman et al, 1979). Nevertheless, retrospective analysis of the use of nitroaromatics in radiotherapy reported clinical efficacy of nitroimidazoles in the treatment of head and neck and bladder cancer (Overgaard and Horsman, 1996).

*Received 30 August 1996*
*Revised 10 February 1997*
*Accepted 11 February 1997*

*Correspondence to:* CB Brezden, 610 University Avenue, Rm. #10-716, Toronto, Ontario, Canada M5G 2M9

Another feature of 2-nitroimidazoles is their selective toxicity towards hypoxic cells (Coleman et al, 1988). The ability to radiosensitize hypoxic cells depends on the intact molecule, whereas the cytotoxic effects are dependent on the bioreductive metabolism of the parent compound. This bioreduction occurs enzymatically by one-electron reductions yielding highly reactive toxic intermediates. The focus of this paper is to further understand the mechanism(s) involved in the selective hypoxic cytotoxicity of 2-nitroimidazoles as an aid to future studies of radiosensitizers and hypoxic cell toxins.

This study of the selective hypoxic cytotoxicity of 2-nitroimidazoles uses a chemically simple model 2-nitroimidazole analogue, 1-methyl-2-nitroimidazole ($INO_2$). The mechanism of the selective hypoxic cytotoxicity of $INO_2$ has been previously shown to occur through the bioreduction of the parent compound to the two-electron nitroso (INO) reductive product (Bérubé et al, 1991). Previous work studied the effects of acute hypoxic exposure to low (2.5 mM) and high (40 mM) $INO_2$ concentrations (Brezden et al, 1994). Hypoxic exposure to 40 mM $INO_2$ resulted in severe thiol depletion of both glutathione (GSH) and protein sulphydryls, loss of intracellular calcium homeostasis and membrane blebbing with fragmented chromatin. In contrast, only GSH depletion with no immediate apoptotic morphology was observed following hypoxic treatment with 2.5 mM $INO_2$ at levels equitoxic to the 40 mM treatment. Although apoptotic morphology was observed in the 40 mM hypoxic treatment at 0–24 h after drug exposure, apoptotic DNA fragmentation as measured by DNA gel electrophoresis was not evident, even up to 48 h after exposure. These cellular differences that were observed after exposure to high vs low concentrations of $INO_2$ suggested the possibility of two mechanisms of cytotoxicity.

In the present study, longer times of post-treatment incubation were investigated to understand the biochemical and morphological changes occurring throughout the time course of the clonogenic assay used to evaluate cell survival. It was found that control populations seeded at equivalent cell densities as drug-treated samples grew rapidly to confluency and then died apoptotically. Possible factors involved in this confluency-induced cell death of non-drug-treated cells were investigated and were controlled for in the drug treatment studies. The results with the drug-treated cells indicate that apoptotic cell death occurred in CHO cells after equitoxic exposure to $INO_2$ under hypoxic conditions at both low and high drug concentrations, but the kinetics of this process were slower at the low drug concentration.

### MATERIALS AND METHODS

### Cells and $INO_2$

Cells used for this study were Chinese hamster ovary (CHO) cells, subclone AA8-4, originally obtained from Dr LH Thompson (Lawrence Livermore Laboratories, CA, USA). CHO cells were grown in suspension and in monolayer culture in alpha-modified minimal essential medium ($\alpha$-MEM) supplemented with 10% fetal bovine serum (FBS) (Sigma, St Louis, MO, USA) and had a doubling time of approximately 12 h. $INO_2$ was synthesized by Dr RA McClelland at the Department of Chemistry, University of Toronto, as previously described by Noss et al (1988). Cellular exposures to $INO_2$ were all performed under hypoxic conditions in suspension culture using procedures previously described by Brezden et al (1994).

For prolonged post-treatment incubation after exposure to either 2.5 mM or 40 mM $INO_2$, cells were counted in a Coulter counter and plated at a cell density of $2 \times 10^6$ cells in 175-cm$^2$ polystyrene flasks (Gibco BRL, Burlington, ON, Canada) and incubated for 1–5 days. Control cell monolayer cultures were seeded for analysis from day 1 to day 5 at the following cell densities: $2 \times 10^6$, $1 \times 10^6$, $5 \times 10^5$, $2 \times 10^5$ and $1 \times 10^5$ cells per flask. This was performed to avoid confluency-induced apoptosis as described below and as seen previously (Brezden and Rauth, 1996). On the desired days, cells were either scraped or trypsinized, resuspended and counted, and prepared for either DNA gel electrophoresis, electron microscopy, propidium iodide (PI) FACS analysis, TdT assay, fluorescence microscopy or alkaline comet assay.

For analysis of the autocrine model of apoptosis, control cells were seeded at $2 \times 10^6$ cells on day 0 and grown to confluency (day 4), at which time cells entered an apoptotic programme. The cultured apoptotic medium (AM) was removed on day 5, filtered and re-added to a subconfluent (day 2) monolayer culture flask for 24 h. Similarly, day 2 subconfluent cultures were either untreated (control) or treated with $\alpha$-MEM supplemented with either 1%, 0.1% or 0% FBS for 24 h. After the 24-h incubation period, cells were harvested as described below for gel electrophoresis.

### Gel electrophoresis and DNA fragmentation

Briefly, after either no drug treatment (control) or an acute 2.5 mM or 40 mM $INO_2$ hypoxic treatment, all cells were collected (adhered and non-adhered) for days 1–5 from the monolayer cultures by either scraping or trypsinization. DNA was isolated and analysed as previously described by Cumano et al (1992) as modified by Brezden et al (1994).

**Figure 1** (**A**) Gel electrophoresis of DNA isolated from CHO cells days 1–5 after plating at an initial cell density of $2 \times 10^6$ cells per flask (at day 0). The DNA marker used was *γDNA–Hin*dIII. (**B**) Transmission electron microscopy of CHO cells at confluency. CHO cells were harvested either at day 2 after plating, representing a control or subconfluent population, or cells were obtained from a 5-day confluent population. Magnification of both electron micrographs was at $5 \times 10^3$ times and a 1-μm bar is shown on each micrograph

© Cancer Research Campaign 1997

**Figure 2** Gel electrophoresis testing an autocrine model of confluency-induced apoptosis. DNA was isolated from CHO cells treated for 24 h with old AM, fresh AM, 1%, 0.1% or 0% FBS supplemented α-MEM (serum starvation) or control (untreated subconfluent monolayer culture) and was electrophoresed. The lane preceding lane 1 represents φχ 174RF DNA–*Hae*III DNA size marker

### FACS analysis

Cells were harvested as described above for gel electrophoresis and were then stained at 4°C for 1 h with 50 μM PI (Sigma); DNA was analysed by flow cytometry (Beckton-Dickinson) using the protocol of Kastan et al (1991). DNA histogram analysis was performed using the Lysis II computer software.

### Terminal deoxynucleotidyl transferase assay (TdT)

Cells were harvested immediately after a 2-h hypoxic treatment or 1 or 5 days after exposure. Briefly, cells were initially fixed at a cell density of $10^6$ cells $ml^{-1}$ in 4% (v/v) formaldehyde (Sigma) for 15 min on ice. After a wash in cold phosphate-buffered saline (PBS), cells were fixed and stored in cold 70% ethanol at –20°C. On the day of the TdT assay, an aliquot was removed that contained $5 \times 10^5$ cells and was put into an eppendorf tube that was precoated with 10% bovine serum albumin (BSA) (Sigma). The cells were centrifuged (240 ***g***) and washed with PBS containing 1% BSA. The pellet was then resuspended in the purchased TdT reaction buffer (Boehringer Mannheim Biochemicals, Indianapolis, IN, USA) using the procedure of Darzynkiewicz et al (1994). However, 10 μM dTTP (Sigma) was included into the reaction buffer to optimize enzyme activity. The treated cells were incubated for 1 h at 37°C and then 1 ml of rinsing buffer (PBS with 0.05% Tween-20, 1% BSA, pH 7.8) was added. The cells were then resuspended in the saline citrate buffer containing 10 μg $ml^{-1}$ avidin-FITC (Sigma), incubated for 60 min at room temperature in the dark, washed with rinsing buffer, resuspended in PBS and analysed by flow cytometry.

### Fluorescence microscopy

Cells growing in flasks were harvested at the desired times after drug exposure by initially removing the supernatant containing the non-adhered cells, trypsinizing the flask to remove the adhered population and combining both populations. The fluorescence staining protocol used was as previously described (McGahon et al, 1995). Briefly, the total collected cells were washed with PBS and were resuspended at a desired cell density of $10^5$ cells per 0.5 ml. To this, 20 μl of a 1:1 mixture of ethidium bromide (100 μg $ml^{-1}$, Sigma) and acridine orange (100 μg $ml^{-1}$, Sigma) was added, and the cell suspension was incubated in the dark at room temperature for 5 min. The stained cells were then centrifuged (250 ***g***) for 5 min and resuspended in 0.1 ml of 10% glycerol. The cell suspension was then mounted onto a slide, coverslipped and photographed with a 40 × objective lens using a Leica Wild MPS 48/52 (Leica Canada) fluorescence microscope fitted with a filter combination for reading fluorescein.

### Alkaline comet assay

Immediately after drug treatment, cells were centrifuged at 700 ***g*** and washed with PBS, and the alkaline comet assay was performed using the protocol of Hu et al (1995). For DNA damage analysis, at least 40 cells were selected at random per each experiment and normalized tail moments were measured as described above.

### Transmission electron microscopy

Transmission electron microscopy was performed on confluent or subconfluent CHO cultures of control non drug-treated cells. Cells were collected by trypsinizing the monolayer cultures followed by a wash with cold PBS and then prepared for transmission electron microscopy as previously described by Karnovsky (1964).

### RESULTS

### Control populations reach confluency and cell death occurs apoptotically

In initial studies of DNA fragmentation, control CHO cells were seeded at the same cell density as drug-treated cells ($2 \times 10^6$ cells per flask). The control monolayer cultures proliferated rapidly (doubling time of 12 h) compared with drug-treated cultures and reached confluency 3 days after plating. At confluency, the untreated CHO cells, as a whole, lost cell-cell and cell-ECM (extracellular matrix) contact, lifted off the monolayer substratum and rapidly died, as assessed by trypan blue exclusion (data not shown). DNA ladders were observed in these control cultures starting 1 day after reaching confluency (day 4 to 5 after seeding, Figure 1A). The confluent cultures, which had lost adherence to the substratum, were visualized by transmission electron microscopy and appeared to have lost cytoplasmic volume, leading to cellular and nuclear shrinkage (Figure 1B). Cells isolated from days 4 to 5 showed chromatin condensation, pyknotic nuclei, organelle compaction and loss of microvilli from

 © Cancer Research Campaign 1997

**Figure 3** Gel electrophoresis after hypoxic exposure to 2.5 mM and 40 mM $INO_2$. CHO cells were treated with either 2.5 mM $INO_2$ (6-h treatment) or 40 mM $INO_2$ (2-h treatment), giving equal toxicity, were washed free from drug and were plated at an initial cell density of $2 \times 10^6$ cells per flask (at day 0). DNA was isolated from pretreated CHO cells from day 1 to day 5 after plating and was electrophoresed on a 2% agarose gel prestained with ethidium bromide. The DNA marker used was $\varphi\chi$ 174RF DNA–*Hae*III size marker

**Figure 4** Fluorescence microscopy of drug-treated CHO cells. CHO cells at 5 days after exposure with untreated hypoxic controls (**A**) or treated with 2.5 mM $INO_2$ (**B**) or with 40 mM $INO_2$ (**C**) were stained with a mixture of ethidium bromide and acridine orange. Control cells (**A**) appear large with green stained nuclei and speckles of bright orange, which represent DNA (green fluorescence) and RNA (orange fluorescence). Apoptotic morphology is observed as yellow fluorescence staining of shrunken nuclei (**B**), whereas cellular ghosts devoid of DNA and RNA are primarily observed in (**C**), indicative of necrotic cell death. Magnification of photographs was × 1720 for **A**, **B**, and **C**

the plasma membrane – all features characteristic of cells dying apoptotically. Control cells that were subconfluent and analysed 2 days after plating appeared round with large nuclei containing heterochromatin and evidence of microvilli on the plasma membrane (Figure 1B). As control populations underwent confluency-induced apoptosis, these untreated control cells were seeded at lower cell densities ($2 \times 10^5$ cells per flask) to avoid confluency, and no DNA fragmentation was seen on days 1–5 post plating (data not shown). Importantly, the drug-treated monolayer cultures never attained confluency. The 2.5 mM $INO_2$-treated population doubled in cell number (from $2 \times 10^6$ to $4 \times 10^6$ cells per flask) over 5 days, whereas the cells exposed to 40 mM $INO_2$ did not increase in cell number and remained at the original seeded cell density of $2 \times 10^6$ cells per flask (data not shown).

### Confluency-induced apoptosis may be mediated by an autocrine mechanism

From 1 to 2 days post-confluency, the entire CHO monolayer culture was rapidly lifting off the substratum and all cells appeared to be dying apoptotically. The rapid kinetic behaviour of this confluency-induced apoptosis, which was observed within the entire population, appeared to be mediated by some signal to which all the cells were responding by undergoing apoptosis. To test for such a signal, cultured apoptotic medium (AM) from confluent apoptotic cell cultures (5 days after initial seeding) was removed, filtered and added undiluted to subconfluent CHO cultures that were initially seeded at $2 \times 10^6$ cells per flask. To assess if confluency-induced apoptosis was simply a result of serum starvation, various concentrations of serum supplemented fresh $\alpha$-MEM were also added to subconfluent CHO cultures. Total cell number count, trypan blue exclusion measurement and gel electrophoresis were assayed 24 h after the medium change. Inhibition of CHO cell

© Cancer Research Campaign 1997 

**Figure 5** CHO cell cycle analysis after hypoxic exposure to 2.5 mM $INO_2$ (**A**) and 40 mM $INO_2$ (**B**). Cells were fixed and stained with 50 µM PI and cell cycle analysis was performed by flow cytometry from day 1 to day 5 after exposure. Both figures show representative untreated control populations that were analysed from a 5-day post-exposure hypoxic control monolayer culture. Cell cycle distributions are represented in three-dimensional DNA histograms. The dimensions are *z*-axis: total cell number; *x*-axis: area of mean fluorescence intensity; and *y*-axis: width of mean fluorescence intensity. Statistical values of each cell cycle phase are displayed as percentage values of 10 000 events analysed. Inset: Histograms revealing positive TdT staining, indicative of cells with fragmented DNA. The *x*-axis represents mean fluorescence intensity of streptavidin-FITC and the *y*-axis represents total cell number of 10 000 cells analysed. (**A**) inset represents control (white) and 2.5 mM $INO_2$-treated (black) cells harvested from 5 days after exposure and (**B**) inset represents control (white) and 40 mM $INO_2$-treated (black) cells harvested from 1 day after exposure

 *© Cancer Research Campaign 1997*

growth by approximately 40% relative to the subconfluent control cells was observed in both cases when 100% cultured AM was added, either old (stored at 4°C for 24 h) or fresh (immediately isolated) (data not shown). This inhibition of growth was greater than that observed for the addition of either α-MEM alone (serum starvation) or 1% or 0.1% FBS supplemented α-MEM (data not shown). Well-defined DNA ladders, characteristic of apoptotic cell death, were only observed for the samples to which cultured AM had been added (Figure 2). Faint laddering was observed in the other samples treated with low serum conditions, however there was considerably less fragmentation compared with the cultured AM-treated samples. Addition of 100% cultured AM to subconfluent cultures was essential for the induction of apoptosis, as AM dilutions of 50% with fresh medium did not result in an apoptotic response (data not shown). These results suggest that an apoptosis-promoting factor may be present in cultures of CHO cells that are undergoing apoptosis.

### Differential cell death after hypoxic exposure at low and high $INO_2$ concentrations

Previous studies have suggested that two mechanisms of cell death may be triggered after hypoxic exposure to low (2.5 mM $INO_2$) vs high (40 mM $INO_2$) drug concentrations. The selective hypoxic cytotoxicity was correlated with a redox imbalance resulting from the bioreduction of the parent compound ($INO_2$) to the highly reactive and toxic nitroso (INO) and/or hydroxylamine (INHOH) intermediates (Bérubé et al, 1991; Brezden et al, 1994). The severity of this redox imbalance and loss of intracellular calcium homeostasis were greater at high than at low concentrations of $INO_2$ at equitoxic drug doses. Therefore, it was of interest to dissect the processes that resulted in this differential in drug-induced cell death by studying the events occurring after drug treatment up to the time of a clonogenic cell survival assay. In order to achieve 0.1% cell survival using a colony-forming assay, cells were treated under hypoxia with 2.5 mM (6 h) or 40 mM (2 h) $INO_2$ and were washed free from drug and replated with fresh α-MEM supplemented with 10% FBS. Cells that were treated with 2.5 mM $INO_2$ fragmented their DNA into oligonucleosomal integers (DNA ladders) 4–5 days after treatment, whereas cells treated with 40 mM $INO_2$ revealed no apoptotic DNA ladders up to 5 days after treatment (Figure 3). This suggests that, rather than there being different mechanisms of cell death, different degrees of apoptosis may occur at high and low $INO_2$ concentrations.

### Differential dose-dependent morphological changes

In order to further understand and characterize the morphological changes in drug-induced cell death that were observed at 2.5 mM compared with 40 mM $INO_2$, fluorescence microscopy was performed. Cells were exposed as above to equitoxic doses of 2.5 mM or 40 mM $INO_2$ under hypoxic conditions to produce 0.1% survival. Cells were once again incubated for 1–5 days after exposure in drug-free medium. As apoptotic DNA fragmentation was observed at day 5 after 2.5 mM $INO_2$ hypoxic exposure, cells were analysed after exposure to both drug concentrations at this time point. A combination of ethidium bromide and acridine orange was used to stain the cells, as these fluorescent dyes are able to provide information on early and late stages of apoptosis and/or necrosis (McGahon et al, 1995).

Figure 4B shows that, 5 days after 2.5 mM $INO_2$ hypoxic treatment, cells show evidence of early apoptosis; the cells contain

**Figure 6** Alkaline comet assay measuring DNA damage. Single-strand DNA damage is represented as length of normalized comet tail moment at equitoxic hypoxic treatments with 2.5 mM (6-h exposure) and 40 mM $INO_2$ (2-h exposure) (white bars) and respective untreated hypoxic control (black bars). Error bars represent standard errors of the mean of at least three independent experiments multiple pyknotic nuclei, with fragmented chromatin observed as apoptotic bodies in the nuclei and cytoplasm (stained yellow); some of these cells have also decreased in size compared with the control population (Figure 4A). However, the cells that were treated with 40 mM $INO_2$ under hypoxia display very different morphological changes compared with the 2.5 mM $INO_2$-treated cell population at 5 days after exposure (Figure 4C). Only a few of these cells showed evidence of late stages of apoptosis, whereas the vast majority of cells appeared as cellular ghosts. These latter cells were stained dark green with no presence of DNA or RNA (stained bright green or orange in the control populations respectively, Figure 4A), indicating that the cells had lost intracellular components while maintaining cellular shape. The presence of cellular ghosts has previously been suggested to be remnants of cells that have undergone necrosis (McGahon et al, 1995). The time course for cellular changes was also monitored by fluorescence microscopy, and cells appeared to decrease in fluorescent staining with time commencing from 1 day after exposure to 40 mM $INO_2$ (data not shown), suggesting the onset of necrosis and the appearance of cellular ghosts at day 5 (Figure 4C). However, immediately after 40 mM $INO_2$ hypoxic treatment, evidence of apoptotic morphology has been reported, which included membrane blebbing, pyknotic nuclei, cellular and nuclear shrinkage and chromatin condensation (Brezden et al, 1994). In contrast, the 2.5-mM-treated population appeared to change gradually, as shown by viable staining, with changes to apoptotic morphology from 1 day (data not shown) to 5 days after exposure (Figure 4B).

Taken together, these observations suggest that cells enter an apoptotic programme after equitoxic hypoxic treatment to both 2.5 mM and 40 mM $INO_2$. However, the 40 mM drug concentration results in an 'accelerated' apoptosis that fails to include DNA ladders and, ultimately, because of secondary necrosis, the cells slowly leach out all cellular components. In contrast, cells exposed to 2.5 mM $INO_2$ enter a more gradual, complete apoptotic programme.

© Cancer Research Campaign 1997

### Differential dose-dependent cell cycle progression and DNA fragmentation

Flow cytometric cell cycle analysis was also performed as a function of time after hypoxic exposure to the two drug concentrations to further establish and understand the mechanisms involved in cell death. Cells treated with 2.5 mM $INO_2$ revealed the presence of an apoptotic sub-$G_1$ population that increased in size from 2 to 5 days after exposure (Figure 5A). Cell numbers remained relatively constant in the $G_1$ phase (approximately 50%), followed by decreased amounts in the S- and $G_2$/M phases (Figure 5A). In contrast, 40 mM $INO_2$ hypoxic treatment halted cells in all phases of the cell cycle, and DNA fragmentation was immediately observed 1 day after exposure, as evidenced by the large sub-$G_1$ population containing 53% of the total cell number (10 000 cells) assayed (Figure 5B). Further study of Figure 5B shows that from days 2 to 5 after treatment, all cells had fragmented their DNA, as the sub-$G_1$ population increased from 83% to 100% of the total cell number assayed (10 000 cells). The observed increasing sub-$G_1$ populations in both 2.5 mM and 40 mM $INO_2$-treated cells may have been the result of contributing factors, such as apoptotic bodies that consisted of either fragmented DNA or further DNA degradation by post-apoptotic mechanisms. The terminal deoxynucleotidyl (TdT) assay was performed to assess the amount of DNA fragmentation in the sub-$G_1$ population. Positive labelling of cells, indicative of apoptotic DNA fragmentation, was observed 24 h after exposure to 40 mM $INO_2$, compared with the control population (Figure 5B, inset). The 2.5 mM $INO_2$-treated cells were also labelled positive by the TdT assay, with the strongest labelling observed at 5 days after exposure (Figure 5A, inset). This provides further evidence that the 40 mM $INO_2$-treated cells entered an 'accelerated' apoptotic pathway compared with the 2.5-mM treated cells and that this 'accelerated' apoptosis may be the result of severe drug-induced cellular damage at day 1. As the initial insult with 40 mM was more severe than with the 2.5 mM treatment, the apoptotic response was completed rapidly, and DNA digestion, indicative of secondary necrosis of the in vitro cell culture, was observed 2–5 days after treatment (Figure 5B). In comparison, 2.5-mM $INO_2$ treatment resulted in a slight $G_1$ arrest and an increasing sub-$G_1$ population over 5 days, suggesting that cells had entered the apoptotic programme and were fragmenting their DNA into the nucleosomal ladders that had been observed on day 5 in Figure 3.

### Differences in initial DNA damage after acute hypoxic exposure at low and high $INO_2$ concentrations

The alkaline comet assay was performed to evaluate the extent of total DNA damage after hypoxic drug exposure to 2.5 or 40 mM $INO_2$. Figure 6 reveals that approximately a threefold increase in DNA strand breaks was observed immediately after 40-mM $INO_2$ treatment (2 h) compared with 2.5 mM hypoxic exposure for 6 h. This may suggest that the extent of total DNA damage immediately after drug treatment governs the fate of the cell, such that moderate DNA damage may undergo initial repair processes and allow some cell progression about the cell cycle, whereas severe damage may overwhelm DNA repair and result in more rapid cell death.

### DISCUSSION

The mechanism of the selective hypoxic cytotoxicity of $INO_2$ was investigated further at both low and high drug concentrations since different physiological end points after equitoxic exposure at these two concentrations had been observed previously (Brezden et al, 1994). These end points included different degrees of protein sulphydryl depletion; 40% depletion at 40 mM $INO_2$ and no depletion at 2.5 mM $INO_2$. Intracellular calcium levels increased to lethal values 3–4 h after 40 mM $INO_2$ treatment under hypoxia, while no increase was observed after 2.5 mM $INO_2$ hypoxic exposure for up to 6 h (Brezden et al, 1994). These experiments suggested that two mechanisms of cell death occurred following low and high concentrations of $INO_2$ under hypoxic conditions.

To further elucidate the mechanisms of cell death following hypoxic $INO_2$ treatment, cells were treated with 2.5 mM and 40 mM $INO_2$ and, when the same 0.1% level of cell survival was reached (6 h and 2 h respectively), cells were washed free of drug and were incubated from day 1 to day 5. This prolonged incubation of 5 days after acute drug exposures allowed for the analysis of the time course and the biochemical and morphological characteristics involved in a clonogenic cell survival assay.

### Confluency-induced apoptosis

In the course of performing these experiments, control cells that were seeded in monolayer cultures at the same cell density as drug-treated cells rapidly proliferated and reached confluency on day 3 after plating. At day 4, these confluent cells lost cell–cell and cell–ECM contact and the whole population began lifting off the substratum. When trypan blue exclusion and gel electrophoresis were performed on these control cells at days 4 and 5, cells appeared to die apoptotically. Figure 1A illustrates the characteristic apoptotic DNA laddering that was observed at days 4 and 5 and which corresponds to 1 and 2 days after confluency. Transmission electron micrographs also revealed characteristic features of apoptosis, such as nuclear and cellular shrinkage, organelle compaction and chromatin condensation, compared with the subconfluent control CHO population (Figure 1B). Care was therefore taken to ensure that the control populations were seeded at a lower cell density than the drug-treated cells to avoid confluency-induced apoptosis and also to obtain the same final cell density after treatment in both untreated and treated populations from days 1 to 5.

Because of the rapid kinetics of apoptosis that were observed to occur within the entire population after reaching confluency, studies were initiated to understand the mechanism. To test whether an autocrine factor was present when cell–cell contact was saturated, cultured apoptotic medium (AM) was isolated and added to a subconfluent CHO monolayer flask. Figure 2 illustrates that both old and fresh cultured AM induced apoptosis in subconfluent CHO monolayer cultures and that this was not due to serum deprivation, as low serum conditions produced considerably less DNA fragmentation than the AM treatment. Moreover, addition of conditioned AM resulted in a growth inhibition of 40% relative to the subconfluent control cells, whereas serum depletion did not affect cell proliferation. These data suggest that a diffusible factor(s) may be secreted from confluent cultures as cells die apoptotically and that the kinetics of apoptosis are different in serum-depleted medium. This phenomenon of confluency-induced apoptosis has also been observed in a number of different cell lines but not in non-transformed cell strains (Brezden and Rauth, 1996).

The importance of cell–cell and cell–ECM interactions have been emphasized by several authors as important regulators of cell viability, specifically through intracellular signalling via integrin

© Cancer Research Campaign 1997

receptors and the focal adhesion kinase p125[FAK] (Meredith et al, 1993; Demarcq et al, 1994). Disruption of epithelial cells from the ECM, called 'anoikis', has been shown to induce apoptosis (Frisch and Francis, 1994). Of note, when CHO cells were grown to extremely high cell densities (approximately $10^6$ cells $ml^{-1}$) in a cell suspension spinner flask, apoptotic cell death was not observed (data not shown), suggesting that confluency-induced apoptosis is as a result of altered cell–cell and/or cell–ECM interactions.

It would be of interest to isolate and characterize the diffusible 'death' factor(s) observed at confluency. One such factor has been identified by Hallahan et al (1989), who reported that TNF-$\alpha$ was secreted from human sarcoma cells after cellular exposure to ionizing radiation and that the production of TNF-$\alpha$ enhanced radiation lethality through autocrine and paracrine mechanisms. In contrast to the presence of a 'death-promoting factor', another possibility may be the absence of critical growth factors required for survival. This can be tested by the addition of growth factors to the AM to determine whether the apoptotic response can be either delayed or inhibited. Preliminary experiments showed that once the apoptotic programme was initiated 1 day after confluency in CHO cells, addition of fresh $\alpha$-MEM + 10% FBS only slightly delayed the apoptotic response induced by confluency. In all subsequent experiments on $INO_2$ toxicity, cell density was maintained and/or adjusted to assure that confluency effects were not confounding drug-induced effects.

### $INO_2$-induced apoptosis

Figure 3 illustrates that apoptotic DNA fragmentation occurred in the 2.5 mM $INO_2$-treated population at days 4 and 5 after an acute exposure, whereas no evidence of DNA ladders was observed with the 40 mM $INO_2$ concentration. It was noted that the 2.5 mM $INO_2$-treated sample underwent one population doubling over the 5 days tested, whereas cells treated with 40 mM $INO_2$ did not increase in cell number. This may suggest that cells are required to re-enter the cell cycle to activate the complete apoptotic programme and cleave their DNA into internucleosomal fragments. Therefore, cells treated with 2.5 mM $INO_2$ proliferated and died apoptotically, while the 40 mM $INO_2$-treated sample remained in a non-proliferating or stationary state that did not result in the initiation of nucleosomal DNA fragmentation as measured by gel electrophoresis.

Apoptotic morphological features, including cell and nuclear shrinkage and fragmented chromatin, were also evident at 5 days after exposure to 2.5 mM $INO_2$ (Figure 4B) compared with the 5-day control (Figure 4A). The technique of fluorescence microscopy allowed for visualization of early and late stages of apoptosis (refer to Materials and methods). After 2.5 mM treatment, early stages of apoptosis were seen consisting of shrunken and pyknotic nuclei that were stained yellow. In contrast, the majority of cells treated with 40 mM $INO_2$ appeared as cellular ghosts devoid of intracellular components, such as RNA and DNA, at day 5 (Figure 4C). These cells were stained dark green, whereas the control cells appeared bright green with bright orange spots indicating DNA and RNA staining respectively (Figure 4A). The presence of cellular ghosts has previously been reported to signify cells that have undergone a necrotic mode of cell death (McGahon et al, 1995).

Analysis of cells immediately after 40 mM $INO_2$ hypoxic exposure (2 h) revealed apoptotic features of cell shrinkage, nuclear compaction and pyknotic nuclei (data not shown). These results were consistent with previous experiments after a 1-h acute hypoxic exposure to 40 mM $INO_2$, which revealed extensive membrane blebbing, nuclear condensation and organelle compaction, as assessed by transmission and scanning electron microscopy (Brezden et al, 1994). This suggests that immediately after the 40 mM $INO_2$ hypoxic treatment, cells have already entered an 'accelerated' apoptotic cell death programme as a result of the severe drug-induced toxic insult. The 'accelerated' apoptotic response fails to induce DNA fragmentation into oligonucleosomal integers, and this ultimately results in random DNA digestion, which represents secondary necrosis. This form of necrosis is an 'artifact' of in vitro cell culture and results from a homogenous cell population undergoing massive cell death. This necrotic response would probably not be observed in vivo because of infiltrating phagocytes and neighbouring cells that would digest and phagocytose the dying apoptotic cells. Different modes of cell death have previously been reported to be induced by varying degrees of the same toxic insult. Dose-dependent cell death has been reported such that low levels of a cellular insult with minor damage to the cells allows the cell to activate the apoptotic programme; whereas at high insult levels the injury to the cell is too severe and the programmed mode of death does not occur, resulting in cell death by necrosis (Fernandes and Cotter, 1994). However, these authors did not comment on whether the observed necrotic cell death was secondary to an 'accelerated' apoptotic response.

Cell cycle analysis after 2.5-mM $INO_2$ hypoxic exposure revealed that the sub-$G_1$ population increased as cells were incubated from day 1 to day 5 after treatment (Figure 5A). Furthermore, as mentioned previously, cells underwent one population doubling from $2 \times 10^6$ cells (initial plating) to $4 \times 10^6$ cells by 4 days after exposure. The presence of an increasing sub-$G_1$ population at day 5 after treatment correlates with the appearance of apoptotic DNA ladders (Figure 3) and apoptotic morphology (Figure 4B). The sub-$G_1$ population observed by FACS analysis may have comprised fragmented DNA due to apoptosis as well as further DNA digestion post-apoptotically and/or drug-induced. The population doubling observed by day 4 (data not shown) immediately before apoptotic DNA fragmentation (Figure 3) is consistent with the requirement of cells initiating the apoptotic cell death programme in an attempt to re-enter mitosis (Pollard et al, 1987; Columbel et al, 1992). In contrast, cell cycle analysis of cells treated with 40 mM $INO_2$ provides evidence that cells attempt to activate apoptosis as observed by the large sub-$G_1$ population at day 1 after acute exposure (Figure 5B). This induction occurs very quickly, which can be attributed to the rapidity of the toxic insult, and as a result random DNA digestion follows and only a sub-$G_1$ signal is observed from day 3 to day 5 (Figure 5B). The presence of an apoptotic sub-$G_1$ population at day 1 supports the model of an 'accelerated' apoptosis followed by a secondary necrotic mode of cell death, however there is no immediate explanation for the absence of DNA ladders at day 1 (Figure 3).

Although DNA ladders were not observed after 40 mM $INO_2$ treatment, DNA fragmentation was evident with the TdT assay. Increased fluorescent labelling, which corresponds to increased apoptotic DNA fragmentation, was observed 24 h after exposure (Figure 5B, inset). Positive TdT labelling was also observed 12 h after hypoxic treatment with 40 mM $INO_2$, even though no DNA ladders were observed when DNA from these treated cells was analysed (data not shown). These results confirm that a dose-dependent apoptotic cell death is observed after treatment with 2.5 and 40 mM $INO_2$, in that an 'accelerated' apoptotic response is

© Cancer Research Campaign 1997

observed at 12–24 h with 40 mM treatment compared with 4–5 days with 2.5 mM treatment.

Figure 6 also provides evidence that more severe DNA damage occurs immediately after 40 mM $INO_2$ exposure compared with an equitoxic 6-h exposure to 2.5 mM $INO_2$. The comet assay provides assessment of DNA strand breaks and an approximately threefold increase in the amount of single-strand DNA breaks was observed at the high 40 mM concentration compared with 2.5 mM. The significant difference ($P < 0.05$) in the total amount of DNA damage observed at the two $INO_2$ concentrations suggests that the extent of total DNA damage governs the initial fate of the cell. Therefore, moderate DNA damage induced by 2.5 mM $INO_2$ treatment may allow the cell to initiate repair processes, and failure of successful DNA repair may lead to the initiation of an apoptotic pathway. In contrast, extensive DNA damage, such as that observed after 40-mM $INO_2$ treatment, may lead to an initial attempt to repair these lesions, and unsuccessful DNA repair may be followed by rapid commencement of apoptosis, leading to the onset of secondary necrosis, as observed by the cellular ghosting mechanism seen in Figure 4C. This observation correlates with the previously reported data that demonstrated different biochemical alterations (protein thiol depletion and $Ca^{2+}$ elevations) observed at equitoxic doses of 40 mM vs 2.5 mM $INO_2$ (Brezden et al, 1994). Whether DNA damage is causal of the kinetics of apoptotic cell death or is a consequence induced by a redox imbalance and loss of intracellular calcium homeostasis after drug treatment (Brezden et al, 1994) remains to be elucidated.

In conclusion, this paper provides evidence that an apoptotic pathway is activated at 2.5 mM $INO_2$ after hypoxic exposure of CHO cells, whereas an equitoxic hypoxic treatment with 40 mM $INO_2$ leads to 'accelerated' apoptosis, rapidly followed by secondary necrosis. Furthermore, the mechanism of confluency-induced apoptosis observed in the control CHO cells has been suggested to occur by an autocrine mechanism, perhaps through the secretion of a 'death' factor(s). These data provide further insight into the mechanism(s) of apoptosis and strongly suggest that proper controls must be pursued when studying the mode of cell death after drug exposure.

### ACKNOWLEDGEMENTS

This work was supported by the National Cancer Institute and the Medical Research Council of Canada. The authors would like to thank P-X Li for help with fluorescence microscopy, A Jang for technical assistance with the comet assay and Y Lin for assistance with the TdT assay. Transmission electron microscopy was performed by B Calvaieri at the Electron Microscopy Unit, University of Toronto, Canada.

### REFERENCES

Bérubé LR, McClelland RA and Rauth AM (1991) Effect of 1-methyl-2-nitrosoimidazole on intracellular thiols and calcium levels in Chinese hamster ovary cells. *Biochem Pharmacol* **42**: 2153–2161

Bleehen NM, Maughan TS, Workman P, Newman HFV, Stenning S and Ward R (1991) The combination of multiple doses of etanidazole and pimonidazole in 48 patients: a toxicity and pharmacokinetic study. *Radiother Oncol* **S20**: 137–142

Brezden CB and Rauth Am (1996) Differential cell death in immortalized and non-immortalized cells at confluency. *Oncogene* **12**: 201–206

Brezden CB, McClelland RA and Rauth AM (1994) Mechanism of the selective hypoxic cytotoxicity of 1-methyl-2-nitroimidazole. *Biochem Pharmacol* **48**: 361–370

Coleman CM, Bump EA and Kramer RA (1988) Chemical modifiers of cancer therapy. *J Clin Oncol* **6**: 709–733

Columbel M, Olsson CA, Ng PY and Buttyan R (1992) Hormone-regulated apoptosis results from re-entry of differentiated prostate cells onto a defective cell cycle. *Cancer Res* **52**: 4313–4319

Cumano A, Paige CJ, Iscove NN and Brady G (1992) Bipotential precursors of B cells and macrophages in murine fetal liver. *Nature* **356**: 612–615

Darzynkiewicz Z, Li X and Gong J (1994) Assays of cell viability: discrimination of cells dying by apoptosis. *Method Cell Biol* **41**: 15–38

Demarcq C, Bunch RT, Creswell D and Eastman A (1994) The role of cell cycle progression in cisplatin-induced apoptosis in Chinese hamster ovary cells. *Cell Growth Diff* **5**: 983–993

Dische S, Saunders MI and Flockhardt IR (1979) MISO – a drug for trial in radiotherapy and oncology. *Int J Rad Oncol Biol Phys* **5**: 851–860

Fernandes RS and Cotter TG (1994) Apoptosis or necrosis: intracellular levels of glutathione influence mode of cell death. *Biochem Pharmacol* **48**: 675–681

Frisch SM and Francis H (1994) Disruption of epithelial cell-matrix interactions induces apoptosis. *J Cell Biol* **124**: 619–626

Hallahan DE, Spriggs DR, Beckett MA, Kufe DW and Weichselbaum RR (1989) Increased tumor necrosis factor α mRNA after cellular exposure to ionizing radiation. *Proc Natl Acad Sci USA* **86**: 10104–10107

Höckel M, Schlenger K, Mitze M, Schäffer U and Vaupel P (1996) Hypoxia and radiation response in human tumours. *Semin Rad Oncol* **6**: 3–9

Hu Q, Kavanagh M-C, Newcombe D and Hill RP (1995) Detection of hypoxic fractions in murine tumors by comet assay: comparison with other techniques. *Rad Res* **144**: 266–275

Karnovsky MJ (1964) The localization of cholinesterase activity in rat cardiac muscle by electron microscopy. *J Cell Biol* **23**: 217–232

Kastan MB, Onyekwere O, Sidransky D, Vogelstein B and Craig RW (1991) Participation of p53 protein in the cellular response to DNA damage. *Cancer Res* **51**: 6304–6311

Koch CJ and Kruuv J (1971) The effect of extreme hypoxia on recovery after radiation by synchronized mammalian cells. *Rad Res* **48**: 74–85

McGahon AJ, Martin SJ, Bissonnette RP, Mahboubi A, Shi Y, Mogil RJ, Nishioka WK and Green DR (1995) The end of the (cell) line: methods for the study of apoptosis *in vitro*. Methods Cell Biol **46**: 153–185

Meredith JE, Fazeli B, and Schwartz MA (1993) The extracellular matrix as a cell survival factor. *Mol Biol Cell* **4**: 953–961

Moulder JE and Rockwell S (1984) Hypoxic fractions of solid tumours: experimental techniques, methods of analysis and a survey of existing data. *Int J Rad Oncol Biol Phys* **10**: 695–712

Noss MB, Panicucci R, McClelland RA and Rauth AM (1988) Preparation, toxicity and mutagenicity of 1-methyl-2-nitrosoimidazole – a toxic 2-nitroimidazole reduction product. *Biochem Pharmacol* **37**: 2585–2593

Overgaard J (1994) Clinical evaluation of nitroimidazoles as modifiers of hypoxia in solid tumors. *Oncol Res* **6**: 509–518

Overgaard J and Horsman MR (1996) Modification of hypoxia-induced radioresistance in tumors by the use of oxygen and sensitizers. *Semin Rad Oncol* **6**: 10–21

Pollard JW, Pacey J, Cheng SV and Jordan EG (1987) Estrogens and cell death in murine uterine luminal epithelium. *Cell Tissue Res* **249**: 533–540

Sutherland RM (1988) Cell and environment interactions in tumor microregions: the multicell spheroid model. *Science* **240**: 177–184

Sutherland RM, Keng PC, Conroy PJ, McDermott D, Bareham BJ and Passalacqua W (1982) *In vitro* hypoxic cytotoxicity of nitroimidazoles: uptake and cell cycle phase specificity. *Int J Rad Oncol Biol Phys* **8**: 745–748

Wasserman DJ, Phillips TL and Johnson RJ (1979) United States clinical and pharmacological evaluation of MISO a hypoxic cell radiosensitizer. *Int J Rad Oncol Biol Phys* **5**: 775–786

Wilson RE, Keng PC and Sutherland RM (1989) Drug resistance in Chinese hamster ovary cells during recovery from severe hypoxia. *J Natl Cancer Inst* **81**: 1235–1240

© Cancer Research Campaign 1997